# miR-122/SIRT1 axis regulates chondrocyte extracellular matrix degradation in osteoarthritis

**DOI:** 10.1042/BSR20191908

**Published:** 2020-06-22

**Authors:** Yinwei Bai, Kun Chen, Jianfeng Zhan, Mingxin Wu

**Affiliations:** Department of Orthopedics, Huizhou Third People’s Hospital, Guangzhou Medical University, Huizhou City 516002, Guangdong Province, P.R. China

**Keywords:** chondrocytes, ECM, miR-122, osteoarthritis, SIRT1

## Abstract

***Background/Aims:*** MicroRNAs (miRNAs) are involved in the pathogenesis of osteoarthritis (OA). The present study aimed to investigate the potential function of miR-122 in the development of OA and its potential molecular mechanisms. ***Methods:*** The expression of miR-122, silent information regulator 1 (SIRT1), collagen II, aggrecan, matrix metalloproteinase (MMP) 13 (MMP13) and ADAMTS4 in OA cartilage was detected by RT-qPCR. Target gene prediction and screening, luciferase reporter assay were used to verify downstream target genes of miR-122. ***Results:*** Compared with osteonecrosis, the expression of miR-122 was significantly increased in OA cartilage, while the expression of SIRT1 was significantly decreased. Overexpression of miR-122 increased the expression of extracellular matrix (ECM) catabolic factors, for example disintegrins, MMPs and metalloproteinases with platelet reaction protein motifs, and inhibited the expression of synthetic metabolic genes such as collagen II and aggregating proteoglycan. Inhibition of miR-122 expression had the opposite effect. Furthermore, SIRT1 was identified as a direct target of miR-122. SIRT1 was significantly inhibited by miR-122 overexpression. Knockdown of SIRT1 reversed the degradation of chondrocyte ECM by miR-122 inhibitors. ***Conclusion:*** The miR-122/SIRT1 axis can regulate the degradation of ECM in OA, thus providing new insights into the treatment of OA.

## Introduction

Osteoarthritis (OA) is a clinically common degenerative disease, mainly characterized by degenerative changes in articular cartilage and subchondral bone, joint pain, difficulty in activities, etc., which occur in middle-aged and elderly people [1,2]. OA is a whole joint involving all joint tissues (cartilage, infrapatellar fat pad, meniscus, synovial membrane and subchondral bone) [3]. The etiology of OA is complex, age, hormone, obesity, joint damage and other factors are involved in the development of OA [4–6]. Cartilage cellularity in OA is reduced by chondrocyte death, and chondrocytes are stimulated by cytokines and growth factors to a catabolic and abnormal differentiation that leads to degradation of the extracellular matrix (ECM) [7–9]. Degradation of the ECM is complicated, for it involves genetic, developmental, biochemical and biomechanical factors. The molecular mechanisms involved in the maintenance of articular cartilage have been characterized in order to develop new therapeutic interventions [10–12].

In the process of OA, pathological changes of cartilage tissue can stimulate the secretion of inflammatory factors [13]. Inflammatory factors can also up-regulate the expression of metabolic proteins such as collagen II, aggrecan, matrix metalloproteinase (MMP) 13 (MMP13) and ADAMTS4 in ECM, leading to metabolic disorders of ECM and promoting the development of OA. The interaction of ECM components forms network structure, which provides nutrition for chondrocyte, and affects various biological functions of chondrocyte through signal transduction system. When OA occurs, the synthesis and degradation of ECM are unbalanced [14,15].

Many microRNAs (miRNAs) are tissue-specific or developmental stage-specific and are associated with diseases such as tumors, lymphomas, viral infections and orthopedic diseases [16]. Recent studies have demonstrated that miRNAs play an important role in the differentiation of osteoblasts and cartilage, and can affect the synthesis and catabolism of osteochondral matrix [17,18]. Studies have shown that many miRNAs play an important role in regulating the development of OA [19]. MiR-122 can participate in the regulation of cell proliferation, programmed death, invasion, metastasis and other life activities. At present, there is no relevant research on the role of miR-122 in OA disease [20,21].

Silent information regulator 1 (SIRT1) is one of the most widely studied members of the sirtuins family. It participates in the pathological process of neurodegenerative diseases, diabetes mellitus, tumors, inflammation, aging and other diseases [22]. In addition, SIRT1 plays an important role in the synthesis of ECM, cell survival and anti-inflammatory effects of human OA chondrocytes [23]. Studies have confirmed that SIRT1expression in OA chondrocytes is lower than that in normal chondrocytes. At the same time, some studies have found that SIRT1-CKO mice are more likely to develop into OA than wild C57BL6/J mice at 8 weeks, accompanied by the increase in collagen X and human MMP13, suggesting that the absence of SIRT1 in chondrocyte accelerates the formation of OA model in mice [24]. But there is no relevant research on the role of miRNA/Sirtl in OA disease. The present study explored the biological role and regulatory mechanism of miR-122 and SIRT1 in OA, providing a new theoretical basis for the treatment and prevention of OA.

## Materials and methods

### Tissue and cell culture

Healthy human articular cartilages from both femoral condyles and tibial plateaus were obtained from victims of road traffic accidents during surgery who had no known history of OA or rheumatoid arthritis (*n*=29; 14 males and 15 females; age range: 50–75 years). Articular cartilage tissues from OA patients undergoing total knee replacement surgery were collected and considered to be the experimental group (*n*=29; 16 males and 13 females; age range: 55–77 years). All studies were approved by the institutional ethics review committee of Huizhou Third People’s Hospital, Guangzhou Medical University. All patients signed a written informed consent form.

SW1353 cells were subcultured in DMEM containing 10% fetal bovine serum. The state of the cells was observed under a microscope, and subculture was performed when the cell density reached 70–80%.

### Quantitative real-time PCR

Total RNA was extracted from cartilage tissue and cells using TRIzol reagent (Invitrogen). After the reverse-transcription reaction, analysis by VIIATM 7 Real-time PCR System. The total RNA was reverse transcribed into cDNA at 37°C using Eastep RT Master Mix kit (Promega Corporation, Madison, WI, U.S.A.), mRNA was quantified using SYBR Premix Ex Taq (Takara Bio, Inc., Otsu, Japan). The thermocycling conditions were as follows: 95°C for 30 s, followed by 45 cycles at 95°C for 5 s, 60°C for 10 s, then 72°C for 20 s. Relative expression was calculated using the 2^−ΔΔ*C*_t_^ method. Glyceraldehyde-3-phosphate dehydrogenase (GAPDH) and U6 were used as internal references. The primers were shown in Table 1.

**Table 1:** The oligonucleotides

Gene	Sense	Antisense
*miR-122*	TATCGCCATGATATACGACACAAAC′	GCCGTGGGAGTGGACCATGGT′
*SIRT1*	GTCACACTTACGACAGAGCAGC	TTTCTCCAGTACATACACAAC
*Collagen II*	TCCTCTGCGACGACATAATCTG	GGTTCTCTCTTCGTCCCTTTG
*Aggrecan*	ATGATTGCTCTCGGCTCCCAG	CTGGGGAGCCAGGAGCGAATCAT
*MMP13*	TGACTGGCAAACTTGAGACGATA	AGGGTGTAATCACCATCTGTAG
*ADAMTS4*	CACTTGCGACACGCTGGGTAT	AGGCGGAGGTCTCACAAGARC
*U6*	CGCTTGCGCGACACATATAC	AAATATGACACTCTCACGA
*GAPDH*	CTGGGCCATACTAGACACACC	AAGTGGTCGTTGAGGGCAATG

### Cell transfection

The miR-122-specific small interfering RNA (siRNA) and the negative control siRNA (NC) were purchased from Ribobio Co., Ltd (Guangzhou, China). The overexpression plasmid vector pcDNA3.1-miR-122 was constructed by GeneChem Co., Ltd. Cell transfection was performed using Lipofectamine 2000 reagent (Invitrogen, Carlsbad, CA, U.S.A.).

### Western blot

The transfected SW1353 cells were centrifuged for 5 min after adding cold PBS and washed for three times with PBS. Then they were cleaved at 4°C for 30 min with Nuclear and Cytoplasmic Protein Extraction Kit (China Haimen Beishi Biotechnology Research Institute), and placed at 4°C for 10 min. The protein was quantified with BCA protein detection kit (Beyotime Institute of Biotechnology). A total of 20 μG protein/lane was separated by 10% SDS/PAGE and transferred to PVDF membrane. The membrane was sealed overnight at 4°C with 5% skimmed milk powder, and then it was incubated with anti-SIRT1 (1:1000), collagen II (1:1000), aggrecan (1:1000), MMP13 (1:1000), ADAMTS4 (1:1000) and GAPDH (1:1000) (Abcam, Cambridge, MA, U.S.A.) were added. After incubating overnight, it was incubated with 1:5000 labeled anti-rabbit secondary antibody for 1 h. After that, the gray values of the target bands and the internal reference bands were recorded by ECL chemiluminescence. The experiment was conducted according to the literature method [25].

### Luciferase reporter gene assay

Wild-type miR-122 (miR-122 WT) and 3′-UTR SIRT1 (SIRT1 3′-UTR WT) reporter plasmid or mutant reporter plasmid (miR-122 MT and SIRT1 3′-UTR MT) with pmirGLO fluorescein enzyme vector (Promega, Madison, WI) were constructed. The SW1353 cells were transfected with a wild-type or mutant plasmid luciferase reporter plasmid. The miR-122 mimetic or miRNA mimetic negative control was then transfected into cells using Lipofectamine 2000 (Invitrogen). The experiment was conducted according to the literature method [26].

### Statistical method

The monitoring data were analyzed by SPSS19.0 statistical software. The results of data analysis are expressed as mean ± standard deviation (mean ± SD). The data between the two groups were analyzed by *t* test. Multigroup data analysis was based on one-way ANOVA. LSD test is used for subsequent analysis. *P*<0.05, the difference was significant.

## Results

### miR-122 and SIRT1 expression levels in OA tissues

**Figure 1:**
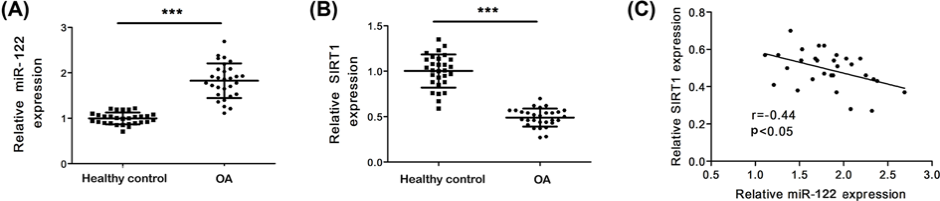
MiR-122 and SIRT1 expression levels in OA cartilage (**A**) miR-122 level in human OA. (**B**) SIRT1 level in human OA. (**C**) Correlation between SIRT1 mRNA expression and miR-122 expression in cartilage samples. Data represent mean ± SD. Compared with the con group, ***P<0.001. con means control.

In order to investigate the functional role of miR-122 in OA, the expression of miR-122 in tissues of OA patients was investigated. As shown in Figure 1A, compared with healthy normal tissues, miR-122 expression was significantly up-regulated in OA tissues (*P*<0.001). Next, the expression level of SIRT1 in tissues of OA patients was measured by quantitative real-time PCR (qRT-PCR). The results in Figure 1B showed that compared with healthy normal tissues, SIRT1 expression was significantly down-regulated in OA tissues (*P*<0.001). There was a direct negative correlation between the expression of miR-122 and SIRT1 in OA tissues (r = −0.44, *P*<0.05) (Figure 1C).

### Effect of miR-122 on the degradation of SIRT1 and chondrocyte ECM in SW1353 cells

**Figure 2:**
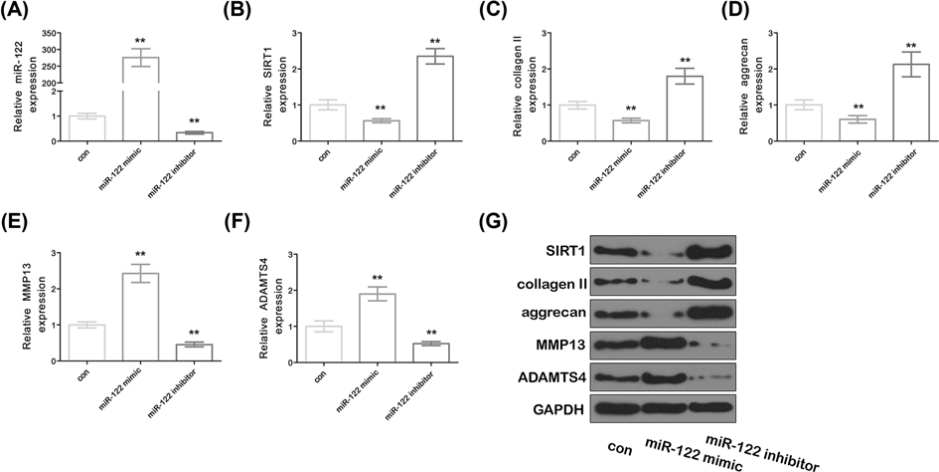
Effect of miR-122 on EMT degradation of SIRT1 and chondrocytes (**A**–**F**) The expression of miR-122, SIRT1, collagen II, aggrecan, MMP13 and ADAMTS4. (**G**) SIRT1, collagen II, aggrecan, MMP13 and ADAMTS4 protein expression were assessed by Western blot. Data represent mean ± SD. Compared with the con group. **P<0.01. con means control.

Real-time PCR results showed that the expression of miR-122 was significantly increased in the miR-122 mimic group compared with that in the control group, while the expression of miR-122 was significantly decreased in the miR-122 inhibitor group (Figure 2A, *P*<0.05). The expression of key proteins collagen II, SIRT1, MMP13, aggrecan and ADAMTS4 of ECM were further analyzed to investigate the effect of miR-122 on the degradation of SIRT1 and chondrocyte ECM. The results were shown in Figure 2B–F. Compared with the control group, the expression of SIRT1, collagen II and aggrecan was significantly decreased in the miR-122 mimic group, while the expression of SIRT1, collagen II and aggrecan was significantly increased in the miR-122 inhibitor group (*P*<0.05) (Figure 2G). Compared with the control group, the expression of MMP13 and ADAMTS4 in the miR-122 mimic group was significantly decreased, while the expression of SMPP13 and ADAMTS4 in the miR-122 inhibitor group was significantly increased (*P*<0.05). The results of the Western Blot further confirmed the above results (Figure 2F).

### SIRT1 was the direct target of miR-122

**Figure 3:**
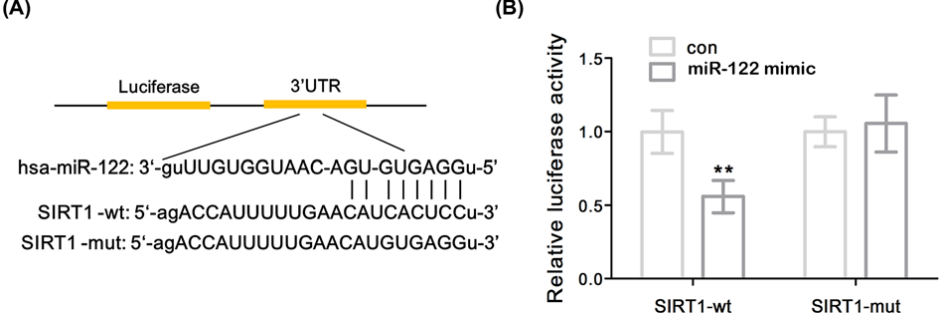
SIRT1 was a direct target of miR-122 (**A**) Schematic representation of the predicted binding site of miR-122 in the 3′-UTR of SIRT1. (**B**) Luciferase activity. Data represent mean ± SD. Compared with the con group, **P<0.01. con means control.

SIRT1 was predicted and identified as a potential target for miR-122 by bioinformatics (Figure 3A). In order to confirm the above experimental results, it was found that the overexpression of miR-122 significantly inhibited the luciferase activity of SIRT1-WT in SW1353 cells by luciferase reporter gene assay (*P*<0.01), while mutations in the 3′-UTR matching site of miR-122 had no significant effect on luciferase activity, suggesting that the interaction between miR-122 and the binding site of the SIRT1 3′-UTR can directly regulate the expression of the luciferase reporter gene (Figure 3B). These results indicated that SIRT1 was able to directly bind to miR-122 and negatively regulate its expression.

### MiR-122 overexpression induced chondrocyte ECM degradation by targeting SIRT1 in SW1353 cells

**Figure 4:**
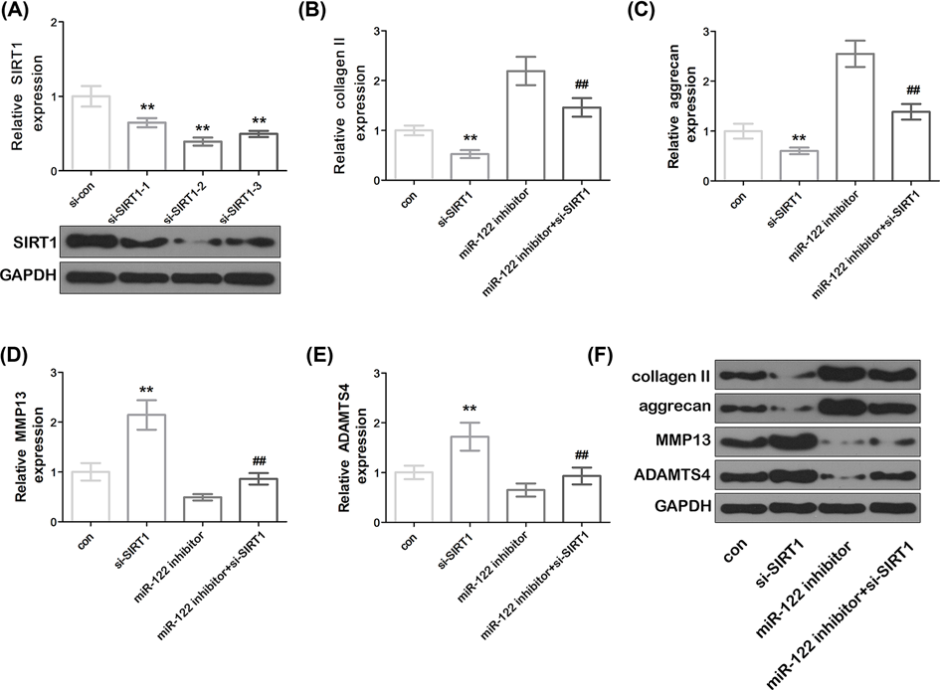
miR-122 induced chondrocyte ECM degradation by modulating SIRT1 (**A**) The mRNA and protein expression levels of SIRT1. Data represent mean ± SD. **P<0.01 compared with si-con. (**B**–**E**) Expression of collagen II, aggrecan, MMP13 and ADAMTS4. (**F**) Expression of collagen II, aggrecan, MMP13 and ADAMTS4 proteins. Data represented mean ± SD. Compared with the con group, **P<0.01, compared with the si-SIRT1 group. ^##^*P*<0.01, compared with the miR-122 inhibitor group. con means control.

The miR-122 inhibitor and the SIRT1 mimetic were co-transfected into the cells to further verify whether miR-122 induces chondrocyte ECM degradation by SIRT1. The knockdown of SIRT1 significantly down-regulated the mRNA and protein expression levels of SIRT1 in cells compared with that in the control group (Figure 4A). As shown in Figure 4B–E, compared with the control group, the knockdown of SIRT1 significantly down-regulated the expression of collagen II and aggrecan mRNA, and significantly increased the expression of MMP13 and ADAMTS4 mRNA in the cells (*P*<0.01). Compared with the control group, the miR-122 inhibitor group was able to reduce the expression level of collagen II and aggrecan mRNA in the cells, and down-regulate the expression levels of MMP13 and ADAMTS4 mRNA. Compared with the miR-122 inhibitor group, knockout of SIRT1 reversed the expression of aggrecan, collagen II, MMP13 and ADAMTS4 mRNA by miR-122 inhibitor. The results of Western blot further confirmed the above results (Figure 4F). The above results indicated that miR-122 can induce chondrocyte ECM degradation by targeting SIRT1.

## Discussion

OA is characterized by articular cartilage degeneration, progressive changes of subchondral bone, osteophyte formation and synovial inflammation, which seriously affects the quality of daily life of patients [27,28]. The most effective treatment so far is only artificial joint replacement [29]. Although it is the most common joint disease in middle-aged and elderly people, the pathogenesis is complicated and it has not yet been clarified. Nowadays, the pathogenesis of OA has become a research hotspot of more and more scholars. Exploring more bone and joint effective treatments for inflammation have also become the focus of the molecular biology field. ECM synthesis and degradation of metabolic disorders are the core steps of OA [30]. Studies have found that the focus of OA cartilage damage is the degeneration of ECM, which is manifested as an imbalance of synthetic degradation [31]. During the degeneration of cartilage ECM, MMPs and their inhibitors, proteoglycans and other proteolytic enzymes play a major role [32]. It has been found that the synthesis of collagen II and proteoglycan is increased in OA patients, but these newly synthesized and their original collagen II and proteoglycan can be over-hydrolyzed by protease, leading to degradation and metabolism far greater than the rate of synthesis and metabolism [33].

We used chondrosarcoma cell line as cellular model, it can replace chondrocytes to a certain extent due to its easy access, strong proliferation ability and stable gene phenotype within a certain regulatory range [34]. Meanwhile, although the cell line is from tumor, it still retains the characteristics of chondrocytes, it can also avoid the possible genotype variation and chondrocytes of primary chondrocytes after long term *in vitro* [35].

Previous studies have revealed that miRNA plays an significant role in the occurrence and development of diseases, and it can provide potential therapeutic targets for diseases [36,37]. MiRNA-146 has been found to be overexpressed in early stage and underexpressed in late stage of OA cartilage, and its decrease is caused by interleukin-Iβ (IL-1β) [38]. It has been found that the age-related proteoglycan loss and fibrosis of articular cartilage in mice after the deletion of miR-140 gene. At the same time, the transgenic mice with high expression of miR-140 had shown resistance to inducing OA. It is also confirmed that miR-140 directly acts on ADAMTS4 and participates in the degradation of cartilage matrix and the development of OA [39]. Based on the complex functions of miRNAs in bone and cartilage, miRNAs are considered to be a novel and efficient method for regulating gene expression in OA.

In the study of the regulation function of miRNAs in bone and cartilage tissues, miR-122 has been found to be a novel factor regulating osteogenic transformation, which inhibits the osteogenic differentiation of mouse myoblasts. In bone cells, miR-122 promotes the development of osteoporosis by inhibiting the activity of osteoblasts and matrix mineralization [40]. However, the function of miR-122 has not been clarified in the mechanism of OA. In this experiment, it is confirmed that miR-122 was down-regulated in OA cartilage. It can be seen that miR-122 may involve in the process of cartilage damage in OA process, accelerate cartilage by regulating cartilage-related degeneration genes. The expression of miR-122 in the cartilage is down-regulated, indicating that miR-122 can play an important role in bone remodeling.

Previous studies have shown that the expression of SIRT1 in OA cartilage were significantly lower than those in normal cartilage, which is consistent with the results of the present study [41]. We screened Sirt1 as a target gene for miR-122, and miR-122 regulated its expression by targeting the 3′UTR of the SIRT1 gene. Furthermore, there was a negative correlation between the expression of miR-122 and SIRT1 in OA cartilage tissue. The above results further confirmed that miR-122 had a direct inhibitory effect on the expression of SIRT1, and miR-122 exerted a biological effect on OA by directly acting on SIRT1.

Some studies have found that the expression of SIRTl in chondrocyte of OA patients is significantly lower than that of normal. After SIRTl-siRNA targeting inhibits the expression of SIRTl, the expression of caspase 3 and 9 in chondrocyte increases, and the apoptotic rate increases accordingly. At the same time, SIRTl-siRNA can up-regulate ADAMTS4, leading to the loss of cartilage matrix. In addition, retroviral-mediated SIRTl plasmid was used to transfect OA chondrocyte, and the expression of SIRT1 increased after overexpression [42]. Our results were consistent with the present study. RT-PCR and Western blot results showed that overexpression of miR-122 can inhibit the expression of SIRT1, collagen II, aggregating proteoglycan, and promote the expression of MMP13 and ADAMTS4, while the inhibition of the expression of miR-122 was contrary to the overexpression of miR-122. This indicated that miR-122 induced ECM damage in chondrocytes. The knockdown of SIRT1 was contrary to the results of miR-122 inhibitors. Compared with the miR-122 inhibitor group, knockout of SIRT1 could reverse the changes of collagen II, aggregating proteoglycan, MMP13 and ADAMTS4 expression treated with miR-122 inhibitor. The above results indicated that miR-122 can induce chondrocyte ECM degradation by targeting SIRT1. This experiment preliminarily explored the role of miR-122 in the pathogenesis of OA, deepened our understanding of this degenerative disease, and provided theoretical basis and action targets for further study of clinical prevention and molecular therapy of OA. In the future, we will use primary chondrocytes isolated from cartilage samples for OA-related research.

## Conclusion

The miR-122/SIRT1 axis can regulate the degradation of ECM of cartilage in OA, which provides a new clue for the pathogenesis of OA and a theoretical basis for the future development of nucleic acid therapy for OA.

## Abbreviations

ECM: extracellular matrix
GAPDH: glyceraldehyde-3-phosphate dehydrogenase
miRNA: microRNA
MMP: matrix metalloproteinase
OA: osteoarthritis
siRNA: small interfering RNA
SIRT1: silent information regulator 1
